# Coexistence of primary aldosteronism and micro-pheochromocytoma: a case report with pathological analysis and literature review

**DOI:** 10.3389/fmed.2026.1864965

**Published:** 2026-07-22

**Authors:** Bo Sun, Hanxiao Yu, Minzhi He, Zhe Wang, Yan Zhang, Ziyu Liu, Jiaming Wen, Xiaoxiao Song

**Affiliations:** 1 Department of Endocrinology, The Second Affiliated Hospital Zhejiang University School of Medicine, Hangzhou, Zhejiang, China; 2 Clinical Research Center, The Second Affiliated Hospital Zhejiang University School of Medicine, Hangzhou, Zhejiang, China; 3 Department of Vascular Surgery, The Second Affiliated Hospital Zhejiang University School of Medicine, Hangzhou, Zhejiang, China; 4 Department of Urology, The Second Affiliated Hospital Zhejiang University School of Medicine, Hangzhou, Zhejiang, China; 5 Department of Pathology, The Second Affiliated Hospital Zhejiang University School of Medicine, Hangzhou, Zhejiang, China

**Keywords:** adrenal nodule, case report, hypertension, micro-pheochromocytoma, pathology, pheochromocytoma, primary aldosteronism

## Abstract

**Background:**

Primary aldosteronism (PA) and pheochromocytoma (PHEO) are both causes of secondary hypertension. Since these two diseases have different pathogeneses, the coexistence of PA and PHEO is very rare and poses challenges in diagnosis and treatment.

**Case presentation:**

A 65-year-old woman developed paroxysmal hypertension two years ago, accompanied by palpitation, dizziness, headache, and vomiting, and she had a poor response to conventional antihypertensive drugs. Contrast-enhanced computed tomography showed a nodule in the medial limb of the right adrenal gland. A mildly elevated aldosterone to renin ratio and an unsuppressed captopril challenge test met the diagnostic criteria for primary aldosteronism. Normetanephrine was elevated in plasma catecholamine testing. Adrenal venous sampling (AVS) demonstrated bilateral aldosterone excess. Right adrenalectomy was performed after multidisciplinary discussion and resulted in rapid clinical improvement. Postoperative pathology revealed coexistence of a cortical aldosterone-producing nodule (APN), a micro-pheochromocytoma and multiple aldosterone-producing micronodules (multiple APM) in adrenal cortex. A *KCNJ5* mutation was found in the APN, representing the first report of this gene mutation in APN in cases coexisting PA and PHEO.

**Conclusions:**

We report a rare case of concurrent PA and micro-pheochromocytoma. This case highlights the importance of early identification and diagnosis, and it provides experience regarding the diagnostic and treatment protocol for such a rare condition. Pathological findings and the *KCNJ5* mutation in APN provide directions for subsequent research on the pathogenesis of concomitant PA and PHEO.

## 1 Introduction

Primary aldosteronism (PA) and pheochromocytoma (PHEO) are both causes of secondary hypertension. Compared with primary hypertension, these two diseases are associated with a higher risk of hypertension complications. PA increases the risk of cardiovascular disease, stoke and renal disease (1–3), and even subclinical PA is negatively associated with cardiovascular health (4). PHEO may cause target tissue damage such as hypertrophic or dilated cardiomyopathy, and lead to fatal cardiac events (5, 6). Since they both need disease-specific therapy, identification and diagnosis are key points in the treatment of the diseases.

About 5 to 14% of individuals with hypertension seen in primary care and up to 30% in referral centers are PA (3), but the morbidity of PHEO is as low as 0.5 per 100,000 people (7, 8). The coexistence of PA and PHEO is very rare and lack of clinical experience. A comprehensive literature review revealed about 20 reported cases worldwide, predominantly involving aldosterone-producing adenomas coexisting with overt pheochromocytomas. The pathogenetic mechanisms underlying this rare coexistence remain poorly understood, with debates centering on whether these dual pathologies arise from independent genetic events or shared developmental pathways.

Here, we report a rare case of concomitant PA and PHEO pathological characterized by the coexistence of an aldosterone-producing nodule (APN) with a micro-pheochromocytoma, accompanied by cortical multiple aldosterone-producing micronodules (multiple APM). This case underscores the importance of comprehensive biochemical screening and offers novel insights into diagnostic workup, surgical strategy, and the genetic mechanisms underlying such rare adrenal pathologies.

## 2 Case report

A 65-year-old woman developed paroxysmal hypertension since 2 years ago, accompanied by palpitation, dizziness, headache and vomiting. The systolic blood pressure fluctuated between 180 and 190 mmHg when the symptoms occurred, and could recover to 130 mmHg without treatment. The symptoms occurred repeatedly without obvious regularity. Levamlodipine 2.5 mg once daily was started 1 year ago for more frequent paroxysmal hypertension, but blood pressure still fluctuated greatly and symptoms did not improve.

**Figure 1:**
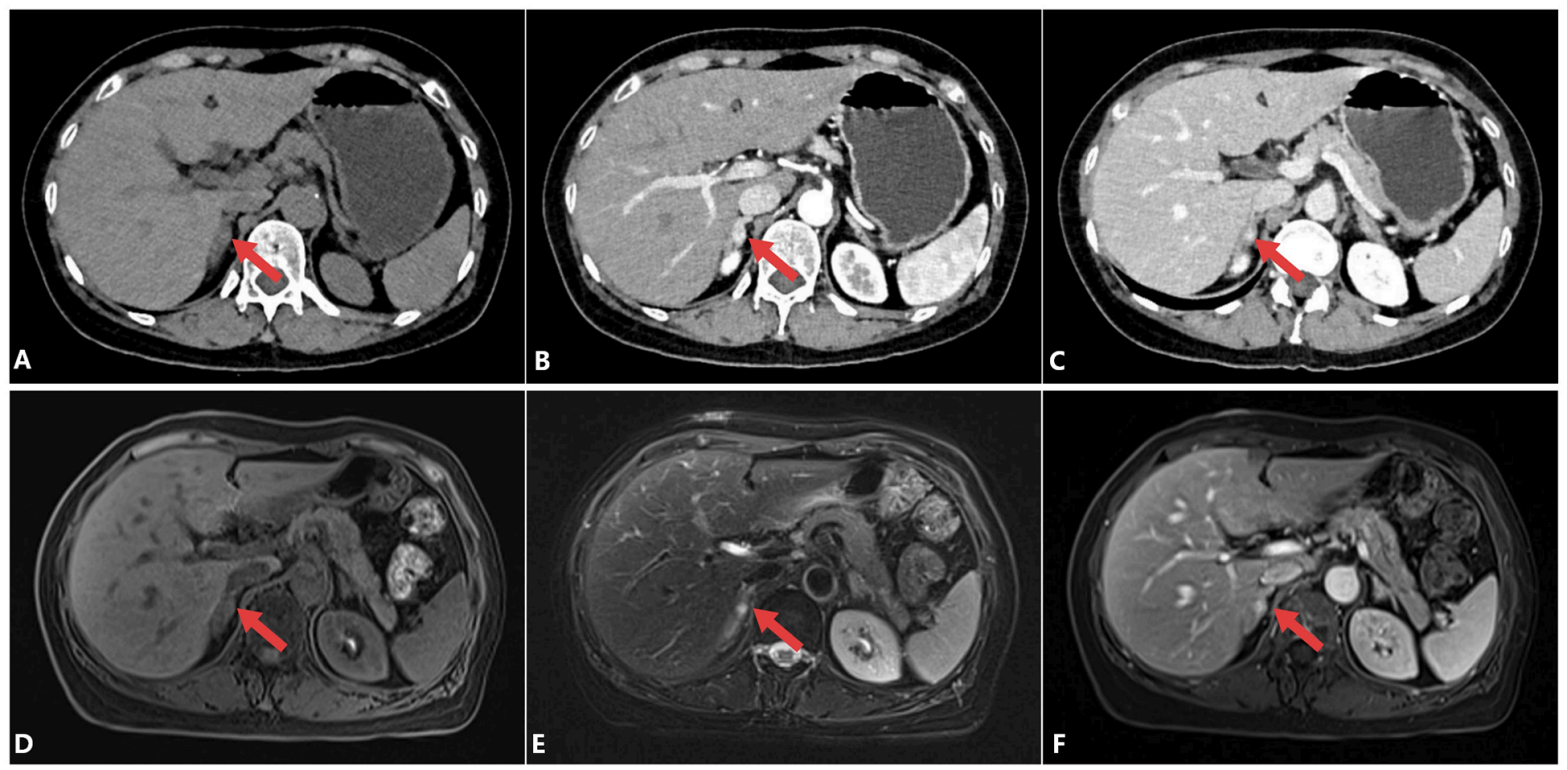
Contrast-enhanced abdominal CT **(A–C)** and contrast-enhanced adrenal MRI **(D–F)** revealed an enhancing right adrenal nodule highly suggestive of pheochromocytoma. Contrast-enhanced abdomen CT **(A**–**C)**: **(A)** unenhanced, **(B)** arterial phase, **(C)** delay phase. Contrast-enhanced adrenal MRI **(D**–**F) (D)** T1 phase, **(E)** T2 phase, **(F)** enhanced phase.

The patient underwent an abdominal contrast—enhanced computed tomography (CT) 20 days ago due to severe vomiting and abdominal distension. The CT showed “a markedly enhancing nodule in the right adrenal gland, suggestive of pheochromocytoma” (Figure 1). After symptomatic treatment, the symptoms of vomiting and abdominal distension improved, and then the patient was transferred to the department of endocrinology for further diagnosis and treatment.

The patient denied a history of hypokalemia and a family history of hypertension. On admission, the patient's height was 156 cm, weight was 51 kg, body mass index (BMI) was 20.96 Kg/m^2^, blood pressure was 156/88 mmHg, heart rate was 82 beats/min, and the rest of the physical examination was normal.

Further evaluation of adrenaline-related hormones (Table 1) showed a normal standing plasma aldosterone concentration (PAC) of 139 pg/mL (normal range 30–353 pg/mL), a suppressed direct renin concentration (DRC) of 3.9 μIU/mL (normal range 4.4–46.1 μIU/mL), an elevated aldosterone to renin ratio (ARR) of 35.64, and a normal potassium level of 3.71 mmol/L (normal range 3.5–5.5 mmol/L). Plasma catecholamines showed an elevated normetanephrine to 378.21 pg/mL (normal range < 164.9 pg/mL), and the other parameters within normal limits. There was no increase in 24-h urinary catecholamines. The levels and diurnal rhythm of serum cortisol and adrenocorticotropic hormone were normal, and sex hormones were normal.

**Table 1:** Initial laboratory results.

Analyte	Result	Normal range
Potassium	3.71	3.5–5.5 mmol/L
Aldosterone	139	30–353 pg/mL
Renin concentration	3.9	4.4- 46.1 μIU/mL
Aldosterone to renin ratio (ARR)	35.64	
Adrenaline	16.74	≤ 141 pg/mL
Metanephrine	20.33	<98.6 pg/mL
Norepinephrine	527.82	70–1700 pg/mL
Normetanephrine	378.21	<164.9 pg/mL
Dopamine	6.94	<30 pg/mL
Vanillylmandelic acid	8.89	<10.11 ng/mL
Cortisol (8 a.m.−4 p.m.−0 a.m.)	307.7–193.0–33.2	166.0–507.0 nmol/L (8 a.m.)
Adrenocorticotropic hormone (8 a.m.−4 p.m.−0 a.m.)	22.1–9.9–6.4	7.2–63.3 pg/mL (8 a.m.)
Testosterone	0.61	<1.58 nmol/L
Androstenedione	2.51	1.00–11.50 nmol/L
Dehydroepiandrosterone sulfate	2.55	0.70–12.39 μmol/L

Due to the elevated ARR, the captopril challenge test was further conducted. The levels of aldosterone and direct renin concentration were 151.0–110.0 pg/mL and 2.9–5.1 μIU/mL (before taking captopril - 2 h after taking captopril), which met the diagnostic criteria of PA in the guidelines (3).

Adrenal contrast-enhanced magnetic resonance imaging (MRI) showed a roundish abnormal signal nodule in the medial limb of the right adrenal gland. The nodule had a smooth margin and was approximately 11 mm × 8 mm in size. It presents as slightly low signal intensity on T1WI and slightly high signal intensity on T2WI. The nodule demonstrates significant enhancement in the arterial phase, portal venous phase, and delayed phase of the enhanced scan (Figure 1).

As the patient considered surgical treatment for PA, adrenal venous sampling (AVS) was necessary. However, when considering the patient's symptoms, imaging findings, and elevated normetanephrine, the patient may have a coexistence of PHEO. We switched the antihypertensive drugs to α-blockade (doxazosin) and volume expansion was performed as preoperative preparation of AVS. We performed non-simultaneous non-ACTH-stimulated AVS; in addition to aldosterone and cortisol, we also collected catecholamines. AVS showed bilateral PA, and catecholamines from the right adrenal gland were not observably higher than those from the left adrenal gland (9–11) (Supplementary Table S1).

We organized a multidisciplinary (MDT) discussion involving the departments of endocrinology, urology, radiology, and laboratory. In combination with the patient's strong surgical willingness to relieve symptoms, a right adrenalectomy was performed after 3 weeks of preoperative preparation.

**Figure 2:**
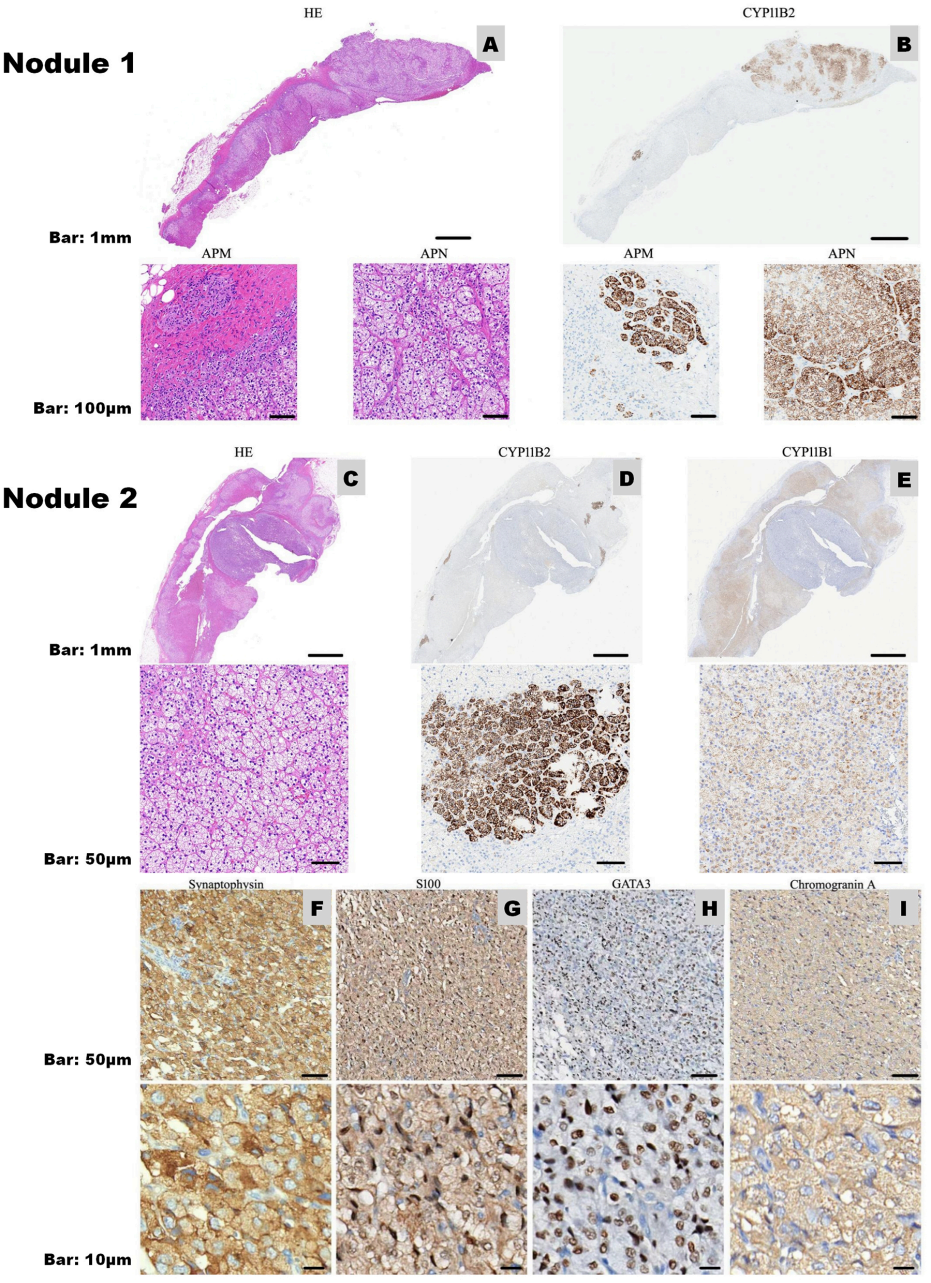
Postoperative histopathological and immunohistochemical findings. Nodule 1 **(A,B)** was identified as an aldosterone-producing nodule (APN), exhibiting focal positivity for CYP11B2 within the nodule and accompanied by aldosterone-producing micronodules (APM). Nodule 2 **(C–I)** was situated in the adrenal medulla and revealed micro-pheochromocytoma with positive immunoreactivity for synaptophysin, chromogranin A, and GATA3. The sustentacular network showed partial staining for S100, consistent with neuroendocrine differentiation. CYP11B2 was negative in Nodule 2 **(D)** but positive in the adjacent adrenal cortex containing multiple aldosterone-producing micronodules (multiple APMs).

Postoperative pathological findings showed (Figure 2): gross examination of the entire right adrenal gland revealed two distinct nodules with a distance of 0.3 cm between them. Due to the limited resolution of imaging modalities, these two nodules do not present distinct boundaries on imaging, and instead manifest as a single nodule with heterogeneous enhancement. Histologically, nodule 1 was consistent with focal features of an aldosterone-producing nodule (APN), characterized by focal positivity for CYP11B2 (aldosterone synthase, approximately 8 × 2.4 mm) within the tumor and negative CYP11B1 (11β-hydroxylase) staining, confirming autonomous aldosterone secretion. The surrounding adrenal cortex showed an aldosterone-producing micronodule (APM) with scattered CYP11B2-positive foci, suggesting a background of nodular cortical hyperplasia. Nodule 2, located in the adrenal medulla and measuring 6.21 × 4.25 mm, demonstrated micro-pheochromocytoma with positive immunoreactivity for synaptophysin, chromogranin A, and GATA3 in the hyperplastic chromaffin cell areas, supporting neuroendocrine differentiation. Irrespective of the size, any distinct nodular proliferation of chromaffin cell origin in the medulla would warrant a diagnosis of micro-pheochromocytoma (7, 12, 13). Within nodule 2 itself, both CYP11B1 and CYP11B2 staining were negative; however, CYP11B1-positive staining and scattered 0.2–1.2 mm CYP11B2-positive foci (multiple aldosterone-producing micronodules, multiple APMs) were observed in the adjacent adrenal cortex surrounding nodule 2. The sustentacular network was partially highlighted by S100 staining, while the low Ki-67 proliferation index (1%), wild-type p53, and negative cyclin D1 indicated a benign hyperplastic process rather than malignancy. SF1 was negative in the medullary component but positive in the surrounding cortex.

We evaluated genetic mutations known to be associated with PA in adrenal gland tissue (*KCNJ5, ATP1A1, ATP2B3, CACNA1D, CTNNB1, CLCN2*) by Sanger Sequencing. A *KCNJ5* mutation, c.451G>A (p.G151R) in exon 2 results in missense mutation, was detected in the APN of nodule 1, whereas no *KCNJ5* or other relevant mutations were identified in the multiple APMs and micro-pheochromocytoma. And we conducted a whole-genome next-generation sequencing of plasma including known genetic mutations associated with PHEO, and no mutation was found.

Postoperatively, the patient reduced the antihypertensive medication by half (levamlodipine 1.25 mg once daily). Blood pressure was controlled at approximately 130/70 mmHg without further fluctuations. Three months after surgery, the potassium level was 4.39 mmol/L (normal range 3.5–5.5 mmol/L), PAC was 149 pg/mL (normal range 30–353 pg/mL), DRC back to normal 8.2μIU/mL (normal range 4.4–46.1 μIU/mL), and ARR was 18.17. Six months after surgery, the potassium level was 4.46 mmol/L (normal range 3.5–5.5 mmol/L), PAC was 81.5 pg/mL (normal range 30–353 pg/mL), DRC was 3.9 μIU/mL (normal range 4.4–46.1 μIU/mL), and ARR was 20.9. Blood catecholamines and their metabolites were within normal ranges (Supplementary Table S2). Antihypertensive medication reduction indicated clinical partial success, normalized ARR but mildly suppressed DRC indicated biochemical partial success according to the Primary Aldosteronism Surgical Outcomes (PASO) criteria (14). Considering that DRC remained mildly suppressed, we switched levamlodipine to spironolactone 10 mg once daily at 6 months postoperatively, and blood pressure was maintained within the normal range. This report has obtained written consent from the patient and was approved by Ethics Committee for Human Research of hospital.

## 3 Discussion

We report a rare case of the coexistence of PA and PHEO. Postoperative pathological found coexistence of cortical APN/APM and micro-pheochromocytoma (7). *KCNJ5* mutation was found in APN, whereas no mutation was detected in micro-pheochromocytoma or multiple APMs. We summarized the clinical and pathological characteristics of cases coexisting PA and PHEO, and conduct a literature review.

### 3.1 Clinical characteristics of cases of concomitant PA and PHEO

The coexistence of PA and PHEO is extremely rare, with only a few reports. Mao et al. reported five cases in the Mayo Clinic from 2000 to 2020 and an additional review of 10 cases from medical literature databases from their inception until November 2019. (15). A retrospective study (2011–2022) reported three PHEO cases with a positive captopril challenge test (16). In addition to these two papers, few more cases were reported (17–20).

Our patient presented with symptoms of catecholamine excess, including headache, palpitations, and marked blood pressure fluctuations, yet serum potassium levels remained consistently normal. This feature is consistent with the three similar cases reported by Mai et al. (serum potassium range: 4.0–4.2 mEq/L) (16) (Supplementary Table S3). However, this differs from the 15-case series summarized by Mao et al. (15), where 87% of patients exhibited hypokalemia and 40% showed symptoms of catecholamine excess. These comparisons suggest that hypokalemia is not a prerequisite when PHEO coexists with PA. Even in the absence of hypokalemia, screening for PA using ARR should be performed to avoid missing a diagnosis of masked aldosteronism.

Only 10 results of AVS have been reported (15, 20). Among these cases, five cases were bilateral and five cases were lateralizing. Confirmed pathologically, there were two cases with ipsilateral PA and PHEO, and three cases contralateral. There is still no report of catecholamine results from AVS. In addition to aldosterone and cortisol, catecholamines were simultaneously collected during the sampling procedure in this case. The result showed that aldosterone secretion was bilateral, and the secretion of catecholamine on the side where the lesion was located was not significantly higher than that on the other side. This may be related to the intermittent or covert secretion of catecholamines.

**Figure 3:**
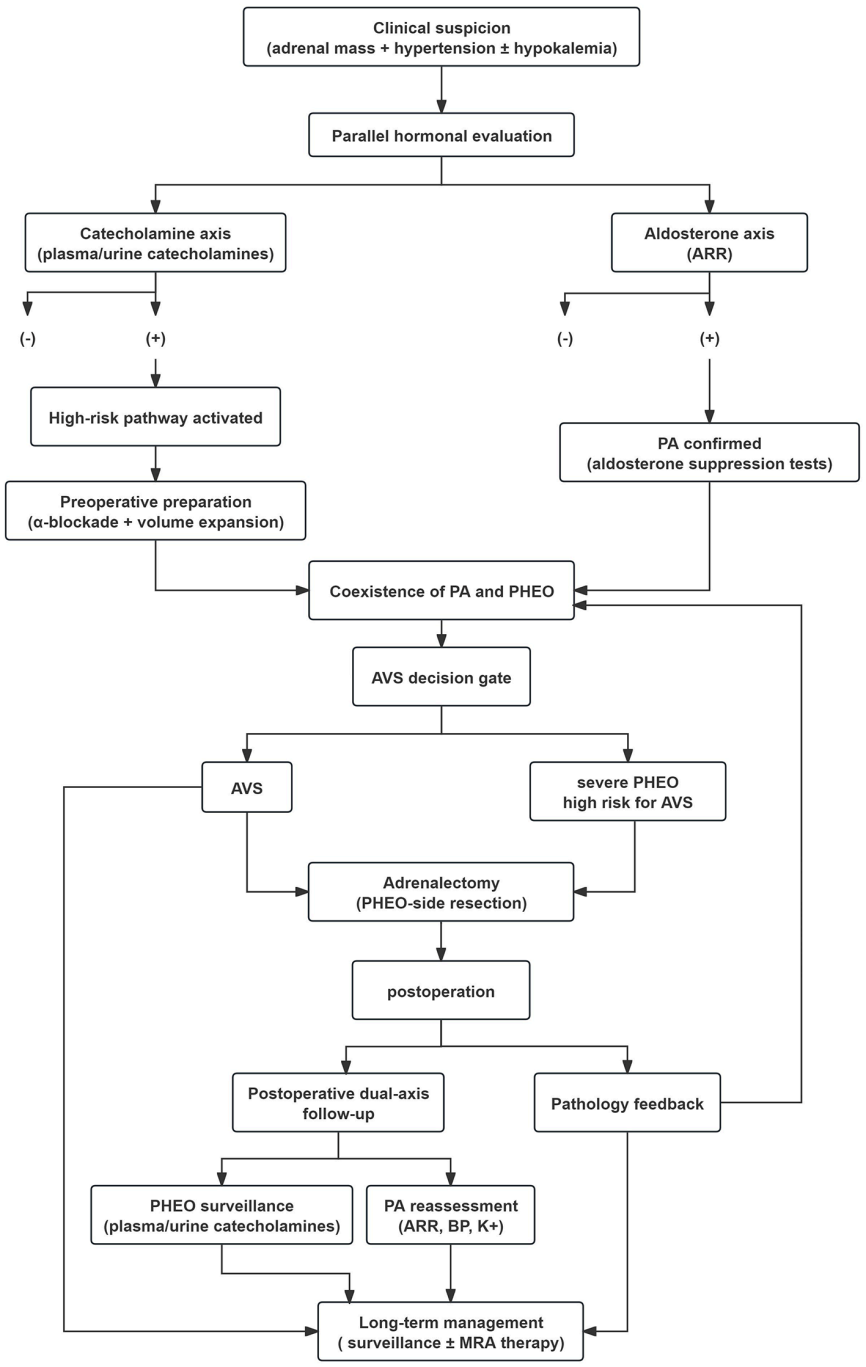
Treatment algorithm for concomitant primary aldosteronism (PA) and pheochromocytoma (PHEO). ARR, aldosterone to renin ratio; MRA, mineralocorticoid receptor antagonist.

The case highlights the importance of early identification and diagnosis, and provides experience about diagnostic and treatment protocol for such a rare condition (Figure 3). The key clinical suspicions for the coexistence of PA and PHEO are adrenal mass and hypertension, with or without hypokalemia. Once there is any suspicion, adrenaline-related hormones should be performed. PA should be confirmed by aldosterone suppression tests and PHEO confirmed by plasma and urine catecholamines. Once PHEO is confirmed, the high-risk pathway should be rapidly activated to avoid a pheochromocytoma crisis. Preoperative preparation (α-blockade and volume expansion) should be performed before AVS or any operation. Compared to PA, PHEO is more severe and life-threatening, and can't be cured by medication. So surgical treatment of pheochromocytoma should take precedence over PA. According to the 2014 Endocrine Society Clinical Practice Guideline, laparoscopic total adrenalectomy is the standard recommendation for most adrenal pheochromocytomas, including unilateral sporadic cases regardless of tumor size (6, 7, 21). AVS can provide a guide for drug choice in long-term postoperative management. If PHEO and PA is ipsilateral, mineralocorticoid receptor antagonists (MRA) is not necessary postoperation, since the lesion of PA has been resected. If PA is contralateral or bilateral, MRA should be used postoperation for the treatment of residual PA. Additionally, for bilateral PA patients with resistant hypertension or heart failure, adrenalectomy can reduce aldosterone burden and improve symptoms of bilateral PA (22, 23). If the patient suffers from severe PHEO with a high risk for AVS, AVS can be skipped. Postoperative dual-axis should be followed. Pathology results can provide feedback on the diagnosis and guide subsequent treatment. For long-term management, surveillance with or without MRA should be performed according to the type of PA and the postoperative symptom remission status.

Importantly, despite the absence of definitive PA functional lateralization in this case, unilateral adrenalectomy targeting the suspected pheochromocytoma-bearing gland resulted in rapid clinical improvement, including resolution of paroxysmal symptoms, stabilization of blood pressure, and a reduction in antihypertensive drug burden. These findings highlight several clinically relevant points. First, micro-pheochromocytoma may exert disproportionate effects on systemic hemodynamics despite minimal or fluctuating biochemical evidence of catecholamine excess. Second, diffuse cortical APMs may represent a subclinical or mild-stage form of PA, which contributes to further blood pressure dysregulation in the background of PHEO without overt biochemical severity, such as hypokalemia. Third, removal of the dominant micro-pheochromocytoma and PA lesion on the same side may sufficiently alleviate neurohumoral instability to achieve meaningful clinical improvement, even in the presence of contralateral residual aldosterone hypersecretion.

### 3.2 Pathological analysis and characteristics of cases of concomitant PA and PHEO

This elderly female patient presented with dual pathological alterations of PHEO coexisting with PA. Postoperatively, her blood pressure stabilized, the pheochromocytoma was cured, and the primary aldosteronism achieved partial remission. The pathological features of this case warrant in-depth discussion from multiple perspectives.

#### 3.2.1 Pathological morphological characteristics based on HISTALDO consensus classification

According to the 2021 International HISTALDO histological consensus criteria (23–25), the right adrenal gland in this case presented three distinct pathological lesions: an aldosterone-producing nodule (APN), multiple aldosterone-producing micronodules (multiple APMs), and micro-pheochromocytoma. CYP11B2, the rate-limiting enzyme for aldosterone synthesis, serves as a key immunohistochemical marker for identifying aldosterone-secreting lesions (26).

##### aldosterone-producing-nodule-apn-with-kcnj5-mutation

Nodule 1 measured 8 × 2.4 mm with patchy CYP11B2 positivity, meeting the diagnostic criteria for APN (morphologically visible lesions < 10 mm with positive CYP11B2 staining, as distinct from APA defined as lesions ≥10 mm) (24). More importantly, this nodule harbored a somatic *KCNJ5* mutation. Current studies indicate that *KCNJ5* mutations represent the most common driver mutations in aldosterone-producing adenoma (APA), with an incidence of approximately 60% in East Asian populations (27), whereas the prevalence of *KCNJ5* mutations is considerably lower in APN. In a cohort of young adults with primary aldosteronism, Nanba et al. reported *KCNJ5* mutations in only 22% (4/18) of APN (28). These mutations promote aldosterone synthesis through loss of potassium channel selectivity, membrane depolarization, and increased calcium influx (29, 30). In this case, the coexistence of focal CYP11B2 positivity and *KCNJ5* mutation is consistent with the immunohistochemical pattern of *KCNJ5*-mutant APA/APN (31). However, CYP11B2 immunohistochemistry may demonstrate significant spatial heterogeneity: approximately 14% of APA cases exhibit intratumoral heterogeneity with coexisting positive and negative regions (32). The “focal positivity” observed in nodule 1 exemplifies this heterogeneity, where positive regions occupied only a portion of the nodule.

##### multiple-aldosterone-producing-micronodules-multiple-apms

Pathological examination revealed multifocal aldosterone-producing micronodules in the right adrenal cortex: scattered CYP11B2-positive foci (long diameter 0.2–1.2 mm) were observed in the adrenal cortical zone, consistent with the HISTALDO consensus definition of APMs (24). Multiple APMs are composed of multiple CYP11B2-positive micronodules (< 10 mm) located beneath the adrenal capsule, interspersed with normal zona glomerulosa. Additionally, the perinodular adrenal cortex surrounding nodule 2 demonstrated CYP11B1 positivity accompanied by scattered CYP11B2-positive foci (long diameter 0.2–1.2 mm), characteristic of APMs immunophenotype. This pattern that CYP11B1/CYP11B2 coexist in adrenal cortex is commonly observed in *KCNJ5*-mutant APA (31). While CYP11B1 converts deoxycorticosterone to corticosterone and theoretically provides substrate for CYP11B2 (33, 34), the functional significance of this phenomenon in APMs requires further investigation. These findings suggest multifocal autonomous aldosterone secretion in this adrenal gland. Moreover, APM may harbor driver mutations identical to APA (such as *CACNA1D, ATP1A1* mutations), but *KCNJ5* mutations are relatively uncommon in APM (35, 36). In this case, no *KCNJ5* mutations were detected in the multiple APMs, consistent with previous studies. The coexistence of *KCNJ5*-mutant APN and mutation-negative APMs in the right adrenal gland suggests a polyclonal, multifocal autonomous aldosterone secretory process.

##### micro-pheochromocytoma

Nodule 2 (6.21 × 4.25 mm) demonstrated positive immunoreactivity for Synaptophysin, Chromogranin A, and GATA3, with partial S100 positivity, consistent with the pathological features of pheochromocytoma. Although Carney et al. defined a nodular lesion of the adrenal medulla smaller than 1 cm as adrenal medullary hyperplasia in 1975 (37), further research found adrenal medullary hyperplasia has the same molecular markers as PHEO, and rename to micro-pheochromocytoma in 2022 (12, 38).

Micro-pheochromocytoma is the early stage or precursor lesion of PHEO, has a significant impact on blood pressure, and should be regarded as PHEO in clinical practice. Sometimes micro-pheochromocytoma may only present refractory hypertension, lack of typical symptoms and slightly elevated or normal plasma/urine catecholamines, which may potentially lead to missed diagnoses (38, 39).

The clinical presentation of PHEO is variable and depends on the tumor size and the secretion type of catecholamines (adrenaline/epinephrine, noradrenaline/norepinephrine and dopamine) (5, 40). This patient had a small size and predominantly noradrenaline-secreting tumor, so the the clinical manifestation was mild (41), but still meet the diagnostic criteria for PHEO. Since catecholamine secretion is pulsatile (5) and we deliberately minimized potential triggers for catecholamine release after we suspected PHEO, no significant release of catecholamines occurred during subsequent urine tests and AVS, resulting in no dominant side being identified.

This nodule tested negative for *KCNJ5* mutation, indicating molecular independence from the cortical lesions.

#### 3.2.2 Comparison of immunohistochemical characteristics and molecular pathology between the present case and previous reports

Compared with previously reported cases of PHEO complicated by PA, the present case shares certain commonalities while also exhibiting several key differences in immunohistochemical features,and molecular pathology (Supplementary Table S4).

In terms of immunohistochemical, the present case is distinguished by a multimodal cortical pathology comprising a solitary, CYP11B2-positive/CYP11B1-negative APN and multifocal APMs with variable CYP11B2 staining. The coexisting micro-pheochromocytoma was negative for both enzymes, confirming its lack of aldosterone synthetic capacity.

This composite pattern differs from prior reports in two respects. First, Mai et al. (16), reported only scattered APCCs without a discrete, *KCNJ5*-mutant APN. Second, Ugi et al. (18), described isolated multiple APMs without a dominant APN. The present case uniquely integrates both features—a *KCNJ5*-driven APN superimposed on mutation-negative APMs—alongside a micro-pheochromocytoma.

In terms of molecular pathology, to our knowledge, this is the first report of a *KCNJ5* mutation in an APN coexisting with PHEO. Mai et al. (16) previously identified the *KCNJ5* (G151R) mutation in PHEO tissues (8.7%), though this gene is generally considered a driver for APA/APN. In this case, the mutation was strictly confined to the APN, with both the micro-pheochromocytoma and multiple APMs being mutation-negative. These findings suggest that *KCNJ5* mutation is unlikely to drive PHEO tumorigenesis but rather represents an independent event in cortical zona glomerulosa-derived APNs.

#### 3.2.3 Pathological explanation for bilateral PA, AVS results, and partial postoperative remission

In this case, adrenal CT and MRI revealed a unilateral nodule; however, AVS indicated bilateral PA, and postoperative pathology confirmed the coexistence of an APN and multiple APMs in the right adrenal cortex. This “imaging-pathology” discrepancy can be understood from the following perspectives.

Multiple APMs may coexist with APA/APN and can contribute to bilateral or diffuse aldosterone secretion abnormalities (24). Multiple APMs (previously defined as aldosterone-producing cell cluster, APCC) typically measure 0.2–1.5 mm in diameter (42, 43), are below the detection limit of CT, as well as undetected APN.

Previous studies have demonstrated that patients with non-classical pathological subtypes of PA often exhibit functional aldosterone secretion from the contralateral adrenal, AVS studies have shown that such patients display significantly higher aldosterone/cortisol ratios in the contralateral adrenal vein (9), which may predict biochemical recurrence during long-term postoperative follow-up.

It is additionally noteworthy that, right adrenal gland had a higher selectivity index (SI) than left adrenal gland (right 49.50 vs. left 2.76), with higher aldosterone (right 4,470 pg/ml vs. left 206 pg/ml) and cortisol (right 13661 nmol/L vs. left 763.3 nmol/L), but lateralization index (LI) was only 1.22 (Supplementary Table S1). This result may be due to our non-simultaneous, non-ACTH-stimulated AVS protocol, a well-established technique acknowledged by international guidelines and a valid alternative to simultaneous sampling. Under unstimulated conditions, physiological pulsatility of cortisol secretion inherently generates inter-side variability (9–11). The lateralization index of 1.22 falled below the unstimulated unilateral cutoff of >2, supporting our bilateral classification independently of absolute SI values. Furthermore, multiple APMs found in postoperative pathological examination and postoperative partial success corroborate bilateral PA independently of the AVS, which is consistent with our AVS results.

Although studies have found some patients with pheochromocytomas and paragangliomas exhibit autonomous cortisol secretion with higher cortisol in peripheral vein (44, 45), PHEO did not alter the results of AVS in previous PA coexisting PHEO case reports (15, 20). These results of AVS were consistent with postoperative pathology and postoperative outcome, regardless of whether PA was ipsilateral, contralateral or bilateral. And to our knowledge, there is still no published evidence about the relationship between PHEO and aldosterone/cortisol in adrenal vein during AVS, which may serve as a potential direction for future research.

Right adrenalectomy removed the *KCNJ5*-mutant APN, APMs, and the micro-pheochromocytoma, thereby correcting catecholamine-mediated blood pressure fluctuations and eliminating partial aldosterone secretory foci. But there was a residual effect of APMs: multifocal APMs exists in the contralateral adrenal tissue, resulting in persistent aldosterone secretion. This pattern of “partial remission” is consistent with postoperative outcomes reported in multiple APMs patients. APMs, due to its multifocal and diffuse nature, often fails to achieve complete biochemical remission following unilateral adrenalectomy (18, 24).

#### 3.2.4 Mechanistic considerations for the coexistence of pheochromocytoma and primary aldosteronism

The coexistence pattern of these two lesions in this case can be analyzed through the following hypotheses.

##### incidental-coexistence

The coexistence of functionally active cortical and medullary tumors within the same adrenal gland is uncommon but well recognized. These collision tumors arise from independent clonal origins, maintaining clear histological boundaries and functional autonomy. Reported cases include hormonally active cortical adenomas—producing either cortisol or aldosterone—alongside pheochromocytomas (13, 15, 16). Such lesions may present as unilateral synchronous masses, involve the contralateral adrenal, or manifest as bilateral cortical micronodular disease with persistent PA after unilateral resection (15, 18, 46). In this patient, the cortical and medullary tumors were clearly separated with no blending, supporting independent origins rather than a common developmental abnormality.

##### common-origin-and-evaluating-the-kcnj5-evidence

Some mixed corticomedullary tumors (MCMT) exhibit intimate cortical-medullary admixture with biphenotypic differentiation, suggesting that they may arise from shared precursors (47, 48). Recent genetic studies challenging the cortical exclusivity of *KCNJ5* mutations may lend further support to this hypothesis. Mai et al. identified *KCNJ5* (G151R) in pheochromocytoma tissues from two of three patients with concomitant PA (16), suggesting a potential shared lineage. However, in our case, the *KCNJ5* mutation was strictly confined to the cortical APN, neither the APM nor the chromaffin cell nodule harbored it. Moreover, the two lesions exhibited mutually exclusive lineage markers: the APN and APMs expressed SF1 and CYP11B2, whereas the chromaffin nodule expressed Synaptophysin, Chromogranin A, and GATA3. The mutually exclusive immunophenotypes, absence of shared driver mutations, and lack of histological intermingling in our specimens strongly favor independent origins. The mechanism underlying *KCNJ5* detection in pheochromocytomas remains to be further investigated.

##### paracrine-interaction

Pheochromocytomas may influence cortical steroidogenesis through multiple mechanisms. Some tumors secrete a variety of peptides, including adrenocorticotropic hormone (ACTH), which could theoretically modulate surrounding cortical cells via local paracrine diffusion through the rich medullary sinusoidal network (46). Conversely, aldosterone may reciprocally influence catecholamine synthesis; in rat pheochromocytoma PC12 cells, aldosterone stimulates dopamine production and upregulates tyrosine hydroxylase mRNA expression via mineralocorticoid receptor activation, an effect blocked by the mineralocorticoid receptor antagonist eplerenone (49). This bidirectional paracrine cross-talk underscores the functional interdependence of cortical and medullary tissues. However, several observations in this case argue against the pheochromocytoma being the primary driver of aldosterone excess. First, the micro-pheochromocytoma was small (6.21 × 4.25 mm), with no histological evidence of cortical hyperplasia in the adjacent zona glomerulosa, suggesting limited local paracrine influence. Second, and most decisively, the patient exhibited only partial remission of primary aldosteronism after unilateral adrenalectomy, with persistent contralateral aldosterone secretion documented by AVS. This clinical course is consistent with bilateral cortical disease rather than a unilateral medullary-driven process.

#### 3.2.5 Clinical implications and the importance of pathological diagnosis

The pathological diagnosis in this case carries critical implications for clinical management.

*Molecular subtyping guides prognosis*: *KCNJ5*-mutant APN typically demonstrates favorable prognosis with high rates of effective postoperative remission. However, the coexistence of multiple APMs and contralateral disease in this case resulted in only partial remission.

*Immunohistochemistry guides surgical strategy*: recognition of APM multifocality may suggest the need for more extensive resection or preparation for postoperative pharmacological therapy.

*Necessity of long-term surveillance*: residual APMs and contralateral disease indicate the need for continued monitoring of ARR and serum potassium, with initiation of mineralocorticoid receptor antagonist therapy when necessary.

In summary, this case, through detailed pathological examination (CYP11B2/CYP11B1 immunohistochemistry, neuroendocrine markers, and molecular testing), reveals a complex pathological landscape of micro-pheochromocytoma coexisting with multifocal primary aldosteronism (APN + APMs), explains the clinical outcome of partial postoperative remission, and emphasizes the central value of precise pathological diagnosis in guiding individualized therapeutic strategies.

## 4 Conclusions

We report a rare case of concomitant PA and micro-pheochromocytoma. This case represents the first report of *KCNJ5*-mutation-positive APN with *KCNJ5*-mutation-negative APMs in concomitant PA and PHEO, and provides additional experience with diagnostic and treatment protocols for such a rare condition. Pathological findings and *KCNJ5* mutation status in the APN provide direction for subsequent research into the pathogenesis of concomitant PA and PHEO.

## Data Availability

The original contributions presented in the study are included in the article/Supplementary material, further inquiries can be directed to the corresponding author.
