# Supplementary material for: Coexistence of primary aldosteronism and micro-pheochromocytoma: a case report with pathological analysis and literature review
Source: Front Med (Lausanne). 2026 Jul 22;13:1864965. doi: 10.3389/fmed.2026.1864965 (PMC13439416; doi:10.3389/fmed.2026.1864965)
Supplement: Supplementary file 1 [file Table_1.docx]

**Supplementary Table 1. Results of unstimulated adrenal venous sampling**

|  | Left adrenal vein | Right adrenal vein | Inferior vena cava(IVC) |
| --- | --- | --- | --- |
| Aldosterone (pg/ml) | 206.0 | 4470.0 | 53.6 |
| Cortisol (nmol/L) | 763.3 | 13661.0 | 276.0 |
| Aldosterone/cortisol | 0.27 | 0.33 | 0.19 |
| Selectivity index (SI) | 2.76 | 49.50 | - |
| Epinephrine (pg/mL) | 10604.70 | 4669.65 | 14.30 |
| Metanephrine (pg/mL) | 4268.40 | 3990.45 | 26.06 |
| Norepinephrine (pg/mL) | 2822.70 | 2717.85 | 273.49 |
| Normetanephrine (pg/mL) | 2050.65 | 2529.60 | 100.40 |
| Dopamine (pg/mL) | 28.81 | 16.80 | 4.32 |
| Vanilmandelic acid (ng/mL) | 10.05 | 9.79 | 9.85 |

Selectivity index (SI): [cortisol]_AV_/[cortisol]_IVC_,

Lateralization index (LI):

([aldosterone]/[cortisol])_highAV_/([aldosterone]/[cortisol])_lowAV_ = 0.33/0.27=1.22

Contralateral suppression index (CSI):

([aldosterone]/[cortisol])_lowAV_/([aldosterone]/[cortisol])_IVC_= 0.27/0.19=1.42

SI>2, Indication of successful AV cannulation

LI =1.22 < 2, and CSI=1.42 >1, Indication of bilateral PA
