# Supplementary material for: Coexistence of primary aldosteronism and micro-pheochromocytoma: a case report with pathological analysis and literature review
Source: Front Med (Lausanne). 2026 Jul 22;13:1864965. doi: 10.3389/fmed.2026.1864965 (PMC13439416; doi:10.3389/fmed.2026.1864965)
Supplement: Supplementary file 2 [file Table_2.docx]

**Supplementary Table 2. Endocrinological findings before and after right adrenalectomy**

| Analyte | Before surgery | After 3 months | After 6 months | Normal range |
| --- | --- | --- | --- | --- |
| Potassium | 3.71 | 4.39 | 4.46 | 3.5-5.5mmol/L |
| Aldosterone | 139 | 149.0 | 81.5 | 30-353pg/mL |
| Direct renin concentration (DRC) | 3.9 | 8.2 | 3.9 | 4.4-46.1 μIU/mL |
| Aldosterone to renin ratio(ARR) | 35.64 | 18.17 | 20.90 |  |
| Adrenaline | 16.74 | 17.65 | 23.82 | ≤141pg/mL |
| Metanephrine | 20.33 | 13.03 | 34.81 | < 98.6pg/mL |
| Norepinephrine | 527.82 | 474.16 | 710.56 | 70-1700pg/mL |
| Normetanephrine | 378.21 | 121.37 | 150.22 | < 164.9pg/mL |
| Dopamine | 6.94 | 5.75 | 1.53 | < 30pg/mL |
| Vanillylmandelic acid | 8.89 | 9.21 | - | < 10.11ng/mL |
