# Supplementary material for: Coexistence of primary aldosteronism and micro-pheochromocytoma: a case report with pathological analysis and literature review
Source: Front Med (Lausanne). 2026 Jul 22;13:1864965. doi: 10.3389/fmed.2026.1864965 (PMC13439416; doi:10.3389/fmed.2026.1864965)
Supplement: Supplementary file 3 [file Table_3.docx]

**Supplementary Table 3. Characteristics and laboratory results in patients of concomitant PA and PHEO with *KCNJ5* mutation**

|  | Case | 1 | 2 | 3 |
| --- | --- | --- | --- | --- |
|  | Sex/Age(years) | M/77 | F/53 | F/65 |
| Pre-operation | | | | |
|  | Blood pressure(mmHg) | 174/89 | 152/88 | 156/88 |
|  | Serum K (mmol/L) | 4.1 | 4.0 | 3.71 |
|  | PAC (pg/mL) | 98 | 74 | 139 |
|  | PRA (ng/mL/h) | 0.9 | 0.2 | - |
|  | DRC (μIU/mL) | - | - | 3.9 |
|  | ARR (PAC/PRA) | 109 | 370 | - |
|  | ARR (PAC/DRC) | - | - | 35.64 |
|  | Captopril challenge test | positive | positive | positive |
| Post-operation | | | | |
|  | Blood pressure(mmHg) | - | - | 130/70 |
|  | Serum K (mmol/L) | - | - | 4.39 |
|  | PAC (pg/mL) | 114 | 65 | 149 |
|  | PRA (ng/mL/h) | 1.0 | 0.7 | - |
|  | DRC (μIU/mL) | - | - | 8.2 |
|  | ARR (PAC/PRA) | 114 | 93 | - |
|  | ARR (PAC/DRC) | - | - | 18.17 |

Case 1 and case 2 are from report in 2025 by Mai.et al. (16)

Case 3 is the patient in our report

PA, primary aldosteronism; PHEO, pheochromocytoma;

K, potassium; PAC, plasma aldosterone concentration; PRA, plasma renin activity; DRC, direct renin concentration; ARR, aldosterone to renin ratio
