# Supplementary material for: Coexistence of primary aldosteronism and micro-pheochromocytoma: a case report with pathological analysis and literature review
Source: Front Med (Lausanne). 2026 Jul 22;13:1864965. doi: 10.3389/fmed.2026.1864965 (PMC13439416; doi:10.3389/fmed.2026.1864965)
Supplement: Supplementary file 4 [file Table_4.docx]

**Supplementary Table 4. Pathology and immunohistochemistry results in patients of concomitant PA and PHEO with *KCNJ5* mutation**

| Case | Sex/Age  (years) | aldosterone-producing nodule (APN) | PHEO tumor tissues and adjacent adrenal glands | *KCNJ5* mutation  lesion location |
| --- | --- | --- | --- | --- |
| 1 | M/77 | No report | CYP11B1 (-) in PHEO tumor, (+) in adjacent adrenal glands  CYP11B2 (-) in PHEO tumor and adjacent adrenal glands  APCC (-) | PHEO tumor tissue |
| 2 | F/53 | No report | CYP11B1 (-) in PHEO tumor, (+) in adjacent adrenal glands  CYP11B2 (-) in PHEO tumor  APCCs (+) in adjacent adrenal glands | PHEO tumor tissue |
| 3 | F/65 | CYP11B1 (-)  CYP11B2 (+)  APM in adjacent adrenal cortex | CYP11B1(-) in PHEO tumor, (+) in adjacent adrenal glands  CYP11B2(-) in PHEO tumor  MAPM(+) in adjacent adrenal glands | APN |

Case 1 and case 2 are from report in 2025 by Mai.et al (16)

Case 3 is the patient in our report

APM, aldosterone-producing micronodule; APCC, aldosterone-producing cell cluster; MAPM,multiple aldosterone-producing micronodules.

APM is formally known as APCC (24)
